# Supplementary material for: Chromatin regulatory dynamics of early human small intestinal development using a directed differentiation model
Source: Nucleic Acids Res. 2021 Jan 6;49(2):726–44. doi: 10.1093/nar/gkaa1204 (PMC7826262; doi:10.1093/nar/gkaa1204)

Supplementary Materials

Supplementary Figure 1. ChRO-seq profiling shows specificity of transcriptional markers in the directed differentiation model of human developing SI. (A) Transcription factors associated with different gastrointestinal organoids that are generated by the hPSC directed differentiation methods. (B) Transcriptional activity (ChRO-seq signal) of genes encoding the transcription factors shown in (A) across different stages. (C) Normalized ChRO-seq signal within the annotated regions of miR-302-607 cluster (minus stranded). Scale (+/- 100 reads/kb/10^6) is fixed across stages. (D) Normalized ChRO-seq signal within the annotated regions of miR-10a (minus stranded). Scale (+/- 25 reads/kb/10^6) is fixed across stages. (E) Characterization of genes with distinct patterns of promoter activity across different stages based on the likelihood ratio test (DESeq2). The protein-coding subset of genes (n = 7,651) were clustered into different groups based on the changing patterns of promoter activity. ChRO-seq study: hESC, n = 3; DE, n = 4; Duo spheroid (Duo), n = 3; Ile spheroid (Ile), n = 3. RNA-seq study: hESC, n = 2; DE, n = 3; Duo, n = 6; Ile, n = 4. TPM, transcripts per million.

Supplementary Figure 2. Steady-state gene expression profiles across all stages in the directed differentiation model of the developing human SI. (A) Hierarchical clustering analysis of RNA-seq expression profiles across stages. Color shade denotes sample to sample distance. (B) PCA of profiles of RNA-seq across stages. (C) RNA-seq expression levels of *SOX2*, *GATA6*, *SOX17*, *CDX2* and *FOXF1* across stages. (D, F, H) Volcano plot showing differentially expressed genes in the indicated comparison. Numbers in red and blue are number of up- and down-regulated genes in the comparison (base mean > 100, log2 fold change of transcription > 1, padj < 0.2 and p < 0.05 by Wald test; DESeq2). (E, G, I) Pathway enrichment analyses of up- and down-regulated genes in the indicated comparisons (GO term = GO Biological Process 2018). hESC, n= 2; DE, n= 3; Duo spheroid (Duo), n = 6; Ile spheroid (Ile), n = 4. nc, normalized counts.

Supplementary Figure 3. Integrative analysis of ChRO-seq and RNA-seq defines marker genes in the directed differentiation model of the developing human SI. (A) RNA-seq expression of genes associated with SI regional identity in spheroids and in HIOs with either Duo or Ile identity. Genes associated with SI regional identity in primary human fetal gut as well as in transplanted HIOs in Tsai et al., 2017. Duo and Ile HIOs were generated by culturing Duo and Ile spheroids in 3D Matrigel for 28 days and purified by EPCAM+ (epithelial marker) sorting. The HIO samples were from the same batch used for ChRO-seq and RNA-seq of Duo and Ile spheroids in this study. (B-E) Genome wide correlation of transcribed levels (ChRO-seq) and expressed levels (RNA-seq) in the indicated stages. No expression or transcription level thresholds were used for genes to be included in the analysis. ChRO-seq study: hESC, n= 3; DE, n= 4; Duo spheroid (Duo), n = 3; Ile spheroid (Ile), n = 3. RNA-seq study: hESC, n= 2; DE, n= 3; Duo, n = 6; Ile, n = 4; Duo HIO, n = 1; Ile HIO, n = 1.

Supplementary Figure 4. ChRO-seq allows identification of active transcriptional regulatory elements (TREs) present in the directed differentiation model of the developing human SI. (A) Size distribution of active TREs. (B) Genomic locations of active TREs (hg38 build).

Supplementary Figure 5. Identification of genes associated with stage-specific enhancers relevant to the stem cell and definitive endoderm stages. (A) Venn diagram showing stage-specific and shared enhancers between hESC and DE. hESC-specific enhancers (n = 3906) were defined in the comparison with DE. (B-C) Cumulative distribution and boxplot of ChRO-seq fold change in the transcriptional activity of genes grouped into three different categories of hESC-specific enhancer density in hESC vs. DE. (D) Identification of genes associated with hESC-specific enhancers (n = 239). (E) Bar graph showing genes associated with hESC-specific enhancers (left panel). Top 30 genes based on enhancer density are highlighted (right panel). (F) Venn diagram showing stage-specific and shared enhancers between hESC and DE. DE-specific enhancers (n = 2899) were defined in the comparison with hESC. (G-H) Cumulative distribution and boxplot of ChRO-seq fold change in transcriptional activity of genes grouped into three different categories of DE-specific enhancer density in DE vs. hESC. (I) Identification of genes associated with DE-specific enhancers (n = 234). (J) Bar graph showing genes associated with DE-specific enhancers (left panel). Top 30 genes based on enhancer density are highlighted (right panel). ChRO-seq study: hESC, n= 3; DE, n= 4. RNA-seq study: hESC, n= 2; DE, n= 3.

Supplementary Figure 6. Identification of hESC- and DE-specific enhancer hotspots and associated genes. (A) hESC-specific stitched enhancers are ranked by transcriptional activity (ChRO-seq signal). The stitched enhancers with the highest transcription activity are defined as hESC-specific enhancer hotspots (n = 211; yellow) and the rest are enhancers non-hotspots (n = 2881; black). (B) ChRO-seq fold change in the transcriptional activity of genes associated with hESC-specific stitched enhancers, non-hotspots vs. hotspots (Wilcoxon test). (C) Identification of genes associated with hESC-specific enhancer hotspots. (D) Genes associated with hESC-specific enhancer hotspots (n=59). Relative position between enhancer hotspots and TSSs of the associated genes are shown. Dot size denotes transcriptional activity of a given DE-specific enhancer hotspot. (E) DE-specific stitched enhancers are ranked by transcriptional activity (ChRO-seq signal). The stitched enhancers with the highest transcription activity are defined as DE-specific enhancer hotspots (n = 147; green) and the rest are enhancer non-hotspots (n = 1987; black). (F) ChRO-seq fold change in the transcriptional activity of genes associated with DE-specific stitched enhancers, non-hotspots vs. hotspots (Wilcoxon test). (G) Identification of genes associated with DE-specific enhancer hotspots. (H) Genes associated with Ile-specific enhancer hotspots (n=46). Relative position between enhancer hotspots and TSSs of the associated genes are shown. Dot size denotes transcriptional activity of a given DE-specific enhancer hotspot. ChRO-seq study: hESC, n= 2; DE, n= 4. RNA-seq study: hESC, n=2; DE, n= 3.

Supplementary Figure 7. Identification of Duo- and Ile-specific enhancer hotspots and associated genes. (A) A Duo-specific enhancer hotspot present around *CDX2* locus. Scale (+/- 100 normalized ChRO-seq reads/kb/10^6) is fixed across stages. (B) A Ile-specific enhancer hotspot present around *HAND2* locus. Scale (+/- 25 normalized ChRO-seq reads/kb/10^6) is fixed across stages. In (A) and (B), the enhancer hotspots defined in this study are overlapped with H3K27ac marked regions of primary human developing SI (the Roadmap Epigenomics Project). The thick blocks denote the individual enhancers within the enhancer hotspots or the narrow H3K27ac peaks within the called gapped regions.

Supplementary Figure 8. TF binding motif enrichment analysis of TREs specific to hESC, DE or Duo spheroids. (A) TF motif enrichment analyses were performed in hESC-specific enhancers (n = 3906) relative to non-hESC-specific TREs (n = 50,777) as well as in hESC-specific enhancer hotspots (n = 211) relative to non-hotspots (n = 2,881). (B) Motifs significantly enriched in hESC-specific enhancers (highlighted by light orange bar) or in hESC-specific enhancer hotspots (highlighted by dark orange bar). The steady-state expression (RNA-seq) of the corresponding TFs are also shown. (C) TF motif enrichment analyses were performed in DE-specific enhancers (n = 2899) relative to non-DE-specific TREs (n = 51,784) as well as in DE-specific enhancer hotspots (n = 147) relative to non-hotspots (n = 1987). (D) Motifs significantly enriched in DE-specific enhancers (highlighted by light green bar) or in DE-specific enhancer hotspots (highlighted by dark green bar). (E) Bubble plot showing full list of genes associated with active binding motifs of TFs that exhibit an overall enrichment of binding motifs in Duo-specific enhancers (defined in Figure 6C). ChRO-seq study: hESC, n= 3; DE, n = 4; Duo spheroid, n = 3.

Supplementary Data 1. Mapping statistics and TRE summary of the ChRO-seq experiments.

Supplementary Data 2. Stage-specific markers at both transcribed and expressed levels.

Supplementary Data 3. Stitched enhancer analysis of the active enhancers specific to hESC, DE, Duo spheroids and Ile spheroids. Parent: the coordinates of a given stitched enhancer; Rank: the ranking based on the Total Signal (ChRO-seq) for a given stitched enhancer; Total Signal: the sum of the ChRO-seq reads from each of the individual enhancers in a given stitched enhancer; NumTRE: the number of individual enhancers within a given stitched enhancer; Length: the length of a given stitched enhancer; Super: denotes whether the Total Signal of a given stitched enhancer is strong enough to be defined as an enhancer hotspot (see Methods); Children: the coordinates of each individual enhancer of a given stitched enhancer.

Supplementary Figure 1.

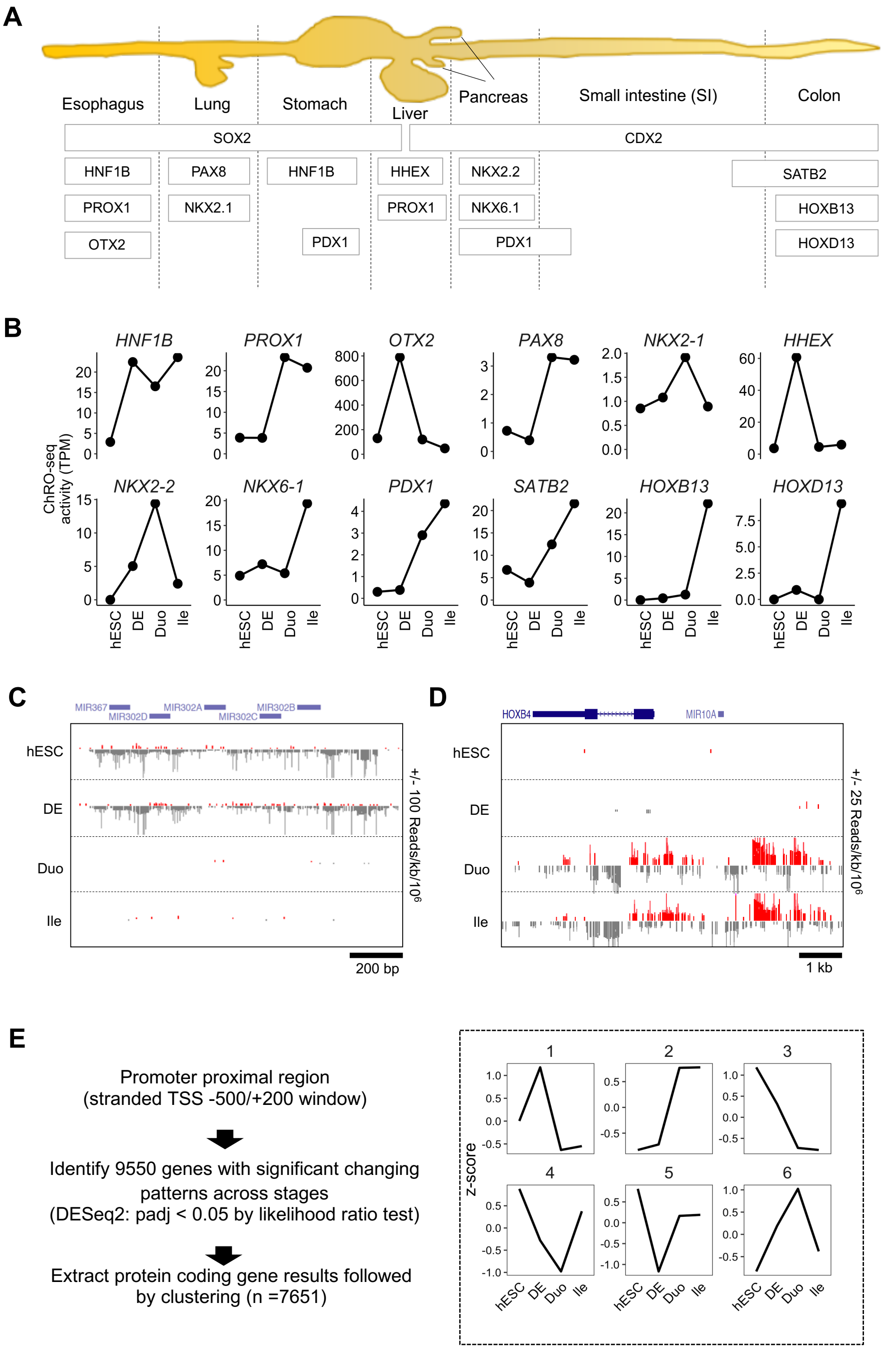

Supplementary Figure 2.

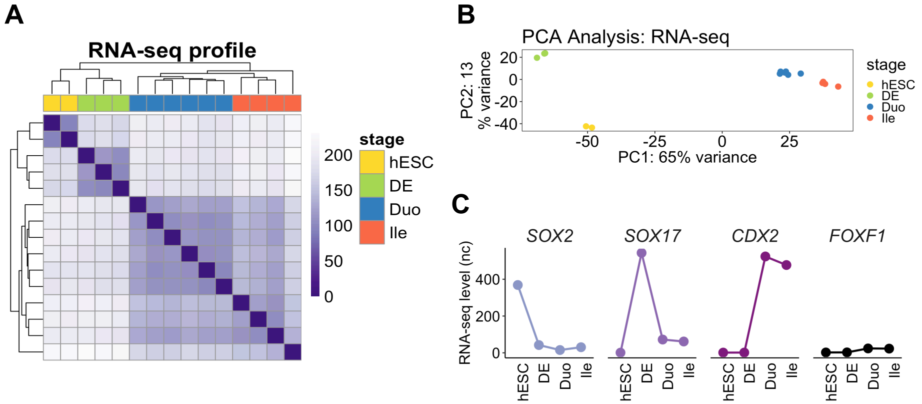

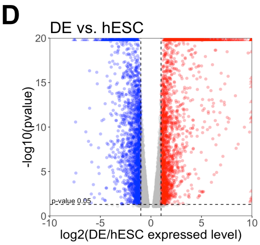

E

GO Term of down-regulated genes in DE vs. hESC	*P* value
Ribosome biogenesis	1.128e-9
rRNA processing	1.448e-8
Carboxylic acid transport	1.804e-8
rRNA metabolic process	6.399e-7
MAPK cascade	9.675e-7

GO Term of up-regulated genes in DE vs. hESC	*P* value
Endoderm formation	1.435e-8
Axon guidance	2.444e-5
ECM organization	5.785e-5
Endoderm development	7.338e-5
Negative chemotaxis	7.637e-5

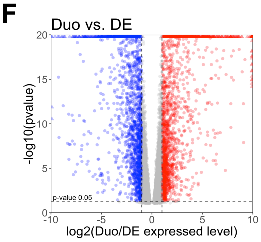

G

GO Term of down-regulated genes in Duo vs. DE	*P* value
Axon guidance	4.775e-8
Axonogenesis	3.226e-6
Apoptosis regulation	4.935e-6
Endoderm formation	1.035e-5
MAPK cascade	6.039e-5

GO Term of up-regulated genes in Duo vs. DE	*P* value
ECM organization	2.958e-18
Kidney development	8.395e-12
Skeletal system development	1.951e-11
Renal system development	5.335e-10
Ureteric bud development	9.528e-10

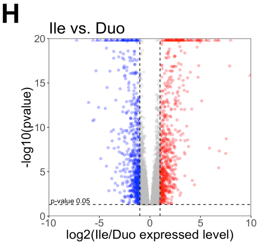

I

GO Term of down-regulated genes in Ile vs. Duo	*P* value
Heart development	1.876e-9
Mesoderm development	1.297e-7
Kidney development	6.208e-7
Circulatory system development	1.831e-6
Renal system development	2.304e-6

GO Term of up-regulated genes in Ile vs. Duo	*P* value
Positive regulation of transcription from RNA pol II promoter	1.042e-12
Positive regulation of DNA-templated transcription	1.388e-12
Regulation of transcription from RNA pol II promoter	1.385e-11
Cell proliferation	1.894e-11
Cell differentiation	2.646e-11

Supplementary Figure 3.

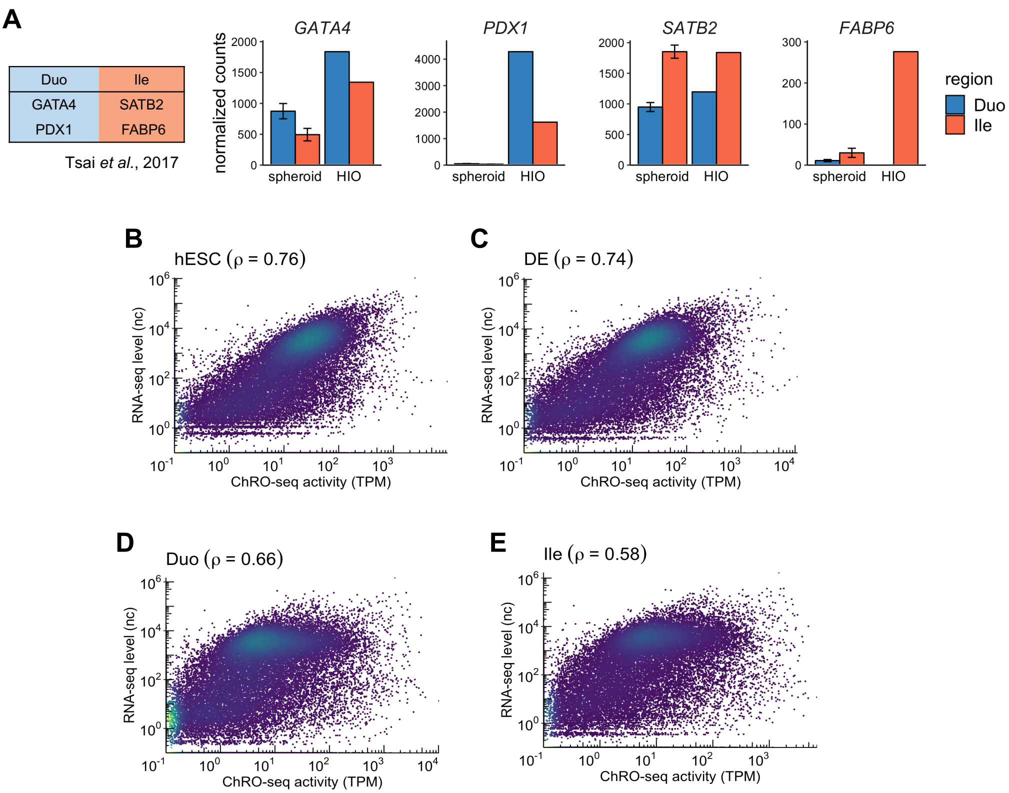

Supplementary Figure 4.

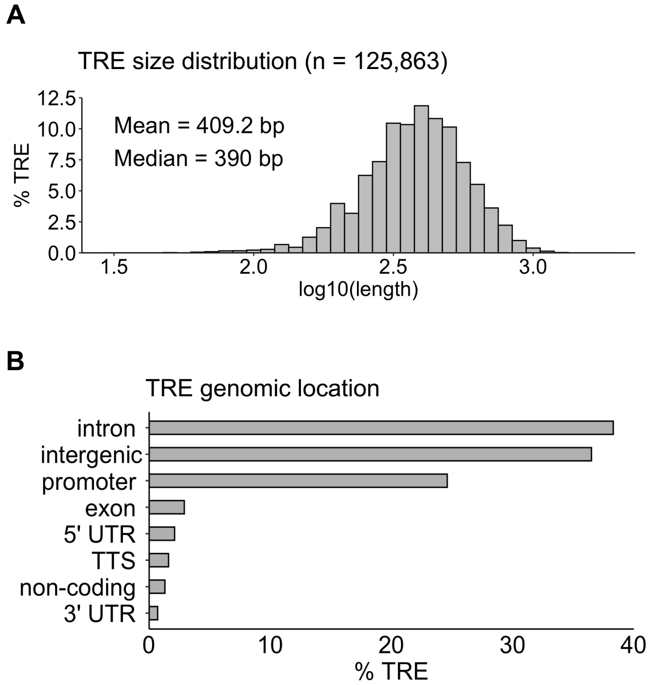

Supplementary Figure 5.

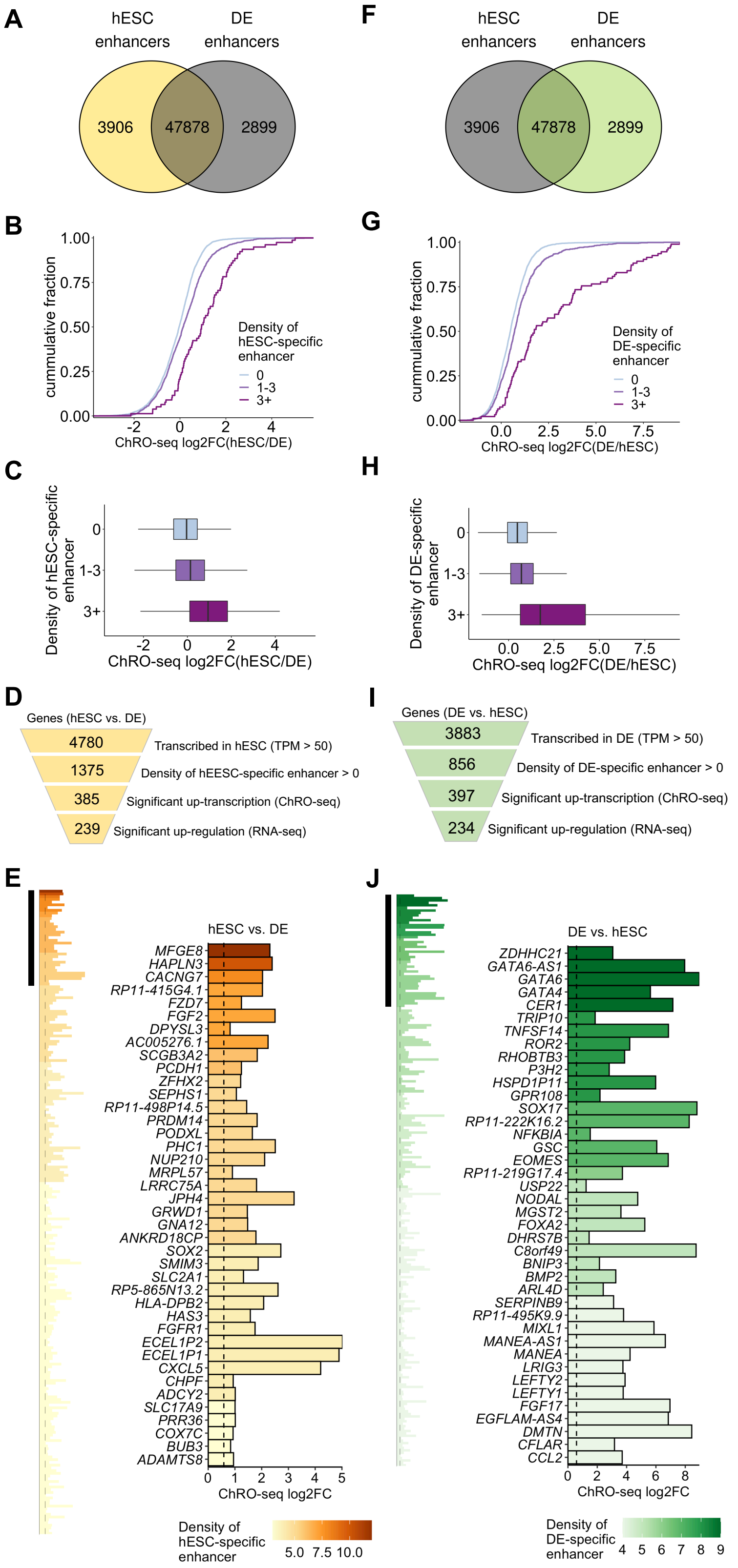

Supplementary Figure 6.

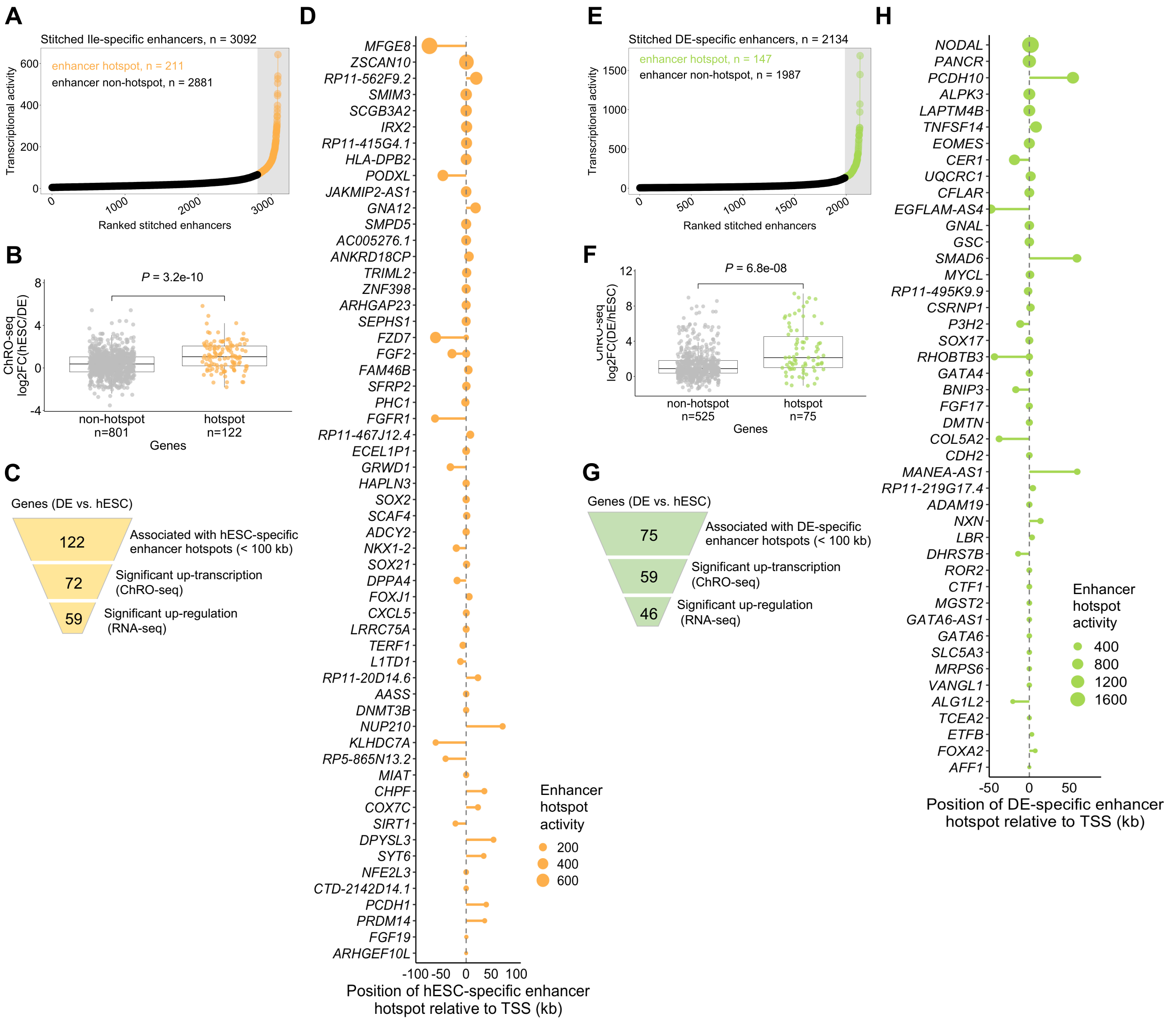

Supplementary Figure 7.

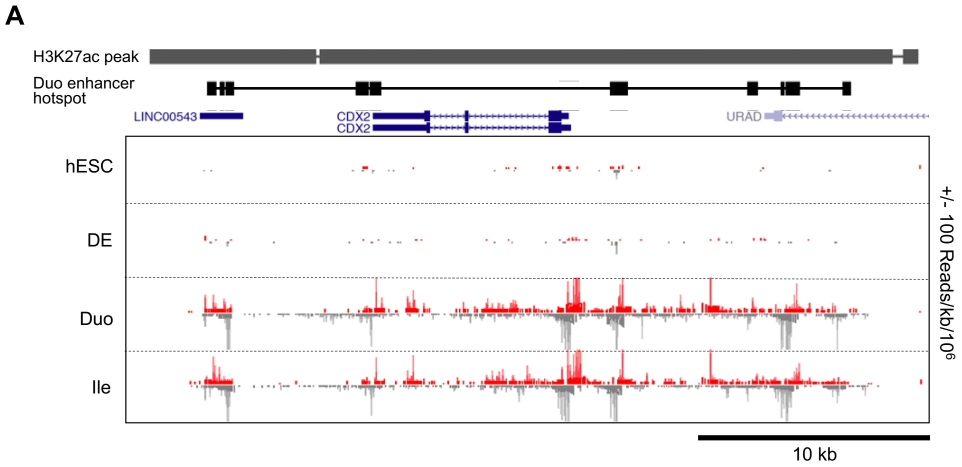

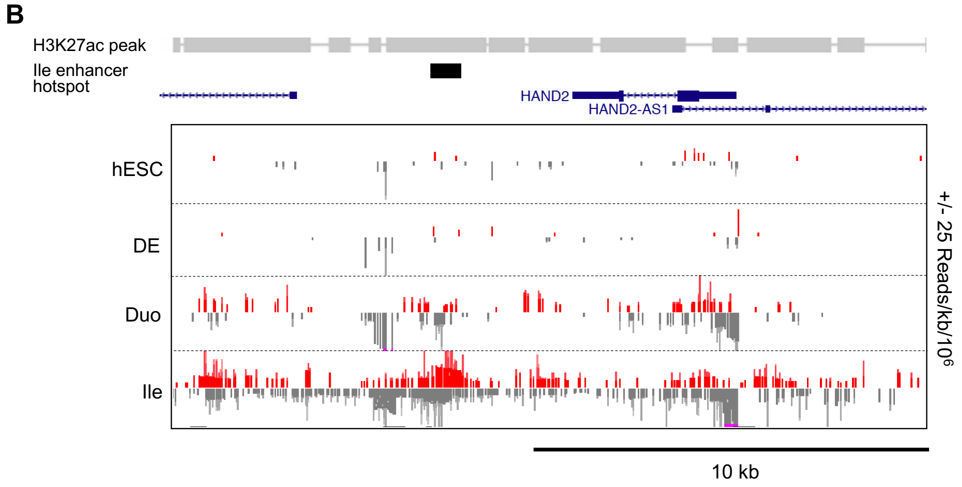

Supplementary Figure 8.

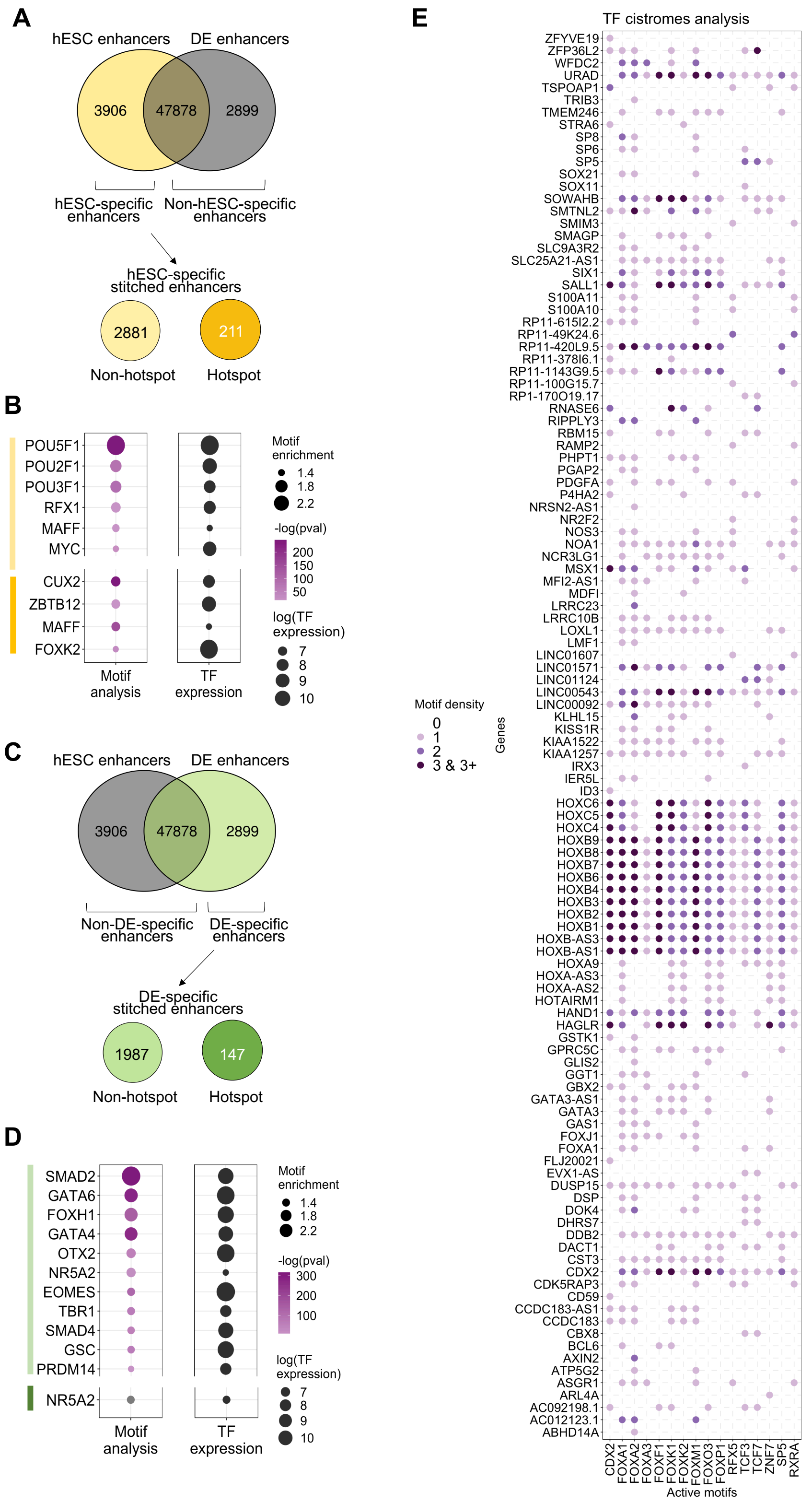

Supplement: gkaa1204_Supplemental_Files [file gkaa1204_supplemental_files.zip › Supplementary_Data_and_Figures_NAR.pdf]
